# Knowledge and Attitudes of Anganwadi Supervisor Workers About Infant (Breastfeeding and Complementary) Feeding in Gondia District

**DOI:** 10.4103/0970-0218.55294

**Published:** 2009-07

**Authors:** Amar Taksande, Satish Tiwari, Alka Kuthe

**Affiliations:** Department of Pediatrics and Mahatma Gandhi Institute of Medical Sciences, Sevagram, Wardha, Maharashtra - 442 102, India; 1 Department of Pediatrics, Punjabrao Deshmukh Medical College, Amravati, Sevagram, India; 2 Department of Obstetric and Gynaecologist, President BPNI, Amravati, Maharashtra State (2007-2008), India

## Introduction

Breastfeeding promotion is a significant child survival strategy. Interventions to improve early and correct infant feeding practices can result in considerable reduction in neonatal morbidity and mortality. A supervisor provides continuous on-the-job guidance to Anganawadi workers (AWWs), to bridge the gap between training and job requirement. Each supervisor looks after 10 Anganawadis and each Anganawadi covers a population of approximately 1000. On her visit to Anganawadi, the supervisor performs certain important tasks like: i) Checks the list of beneficiaries from the low economic strata, who are severely malnourished, ii) Guides AWWs in the assessment of correct ages of children, correct method of weighing the children, and plotting their weights on growth charts, iii) Demonstrates to the AWWs the effective methods of providing health and nutrition education to mothers, and iv) Maintains the statistics of the Anganawadis.(1) Her work includes health and nutrition education on various aspects of the health of a mother and child. Therefore, it is important for the supervisor to have adequate scientific knowledge about infant breastfeeding. This will help her to impart correct knowledge to mother beneficiaries (pregnant, lactating mothers, and women in the reproductive age group).(2,3) The objective of the present study is to assess the knowledge (scientific and latest) and attitude of the Anganawadi supervisors with regard to infant feeding and also to identify gaps in their knowledge.

## Materials and Methods

The infant and young child feeding training program was jointly conducted by the Gondia branch of Breast Feeding Promotion Network of India (BPNI) and Government of India, Maharashtra. Thirty-six Anganawadi supervisors (all of them women) were the participants. They were briefed about the objectives of the study. Before the training program, a pretest questionnaire was administered to the participants. The questionnaire consisted of 20 multiple-choice questions covering issues on the entire IYCF (infant and young child feeding). After the pretest, IYCF multimedia lecture sessions on breast feeding, complementary feeding, and breastfeeding problems were undertaken. The teaching methods adopted were live demonstrations and role-plays. Small group discussions were also conducted after each session. The effectiveness of the training program was assessed by conducting a post-test assessment using the same questionnaire after the training program. Correct responses to test items in the questionnaire were given one mark with a maximum of 20 marks. We divided the participants going by their pre- and post-test scores into ‘very good score’ (if the participant secured ≥18), ‘good score’(15-17) ‘average’(12-14) and ‘below average’ score (≤11). The pre- and post-answer sets were evaluated, marked, and compared. The Statistical Package for Social Science (SPSS) 10 version was used for statistical purposes, and calculated the t test statistic for paired samples.

## Results

**Figure 1:**
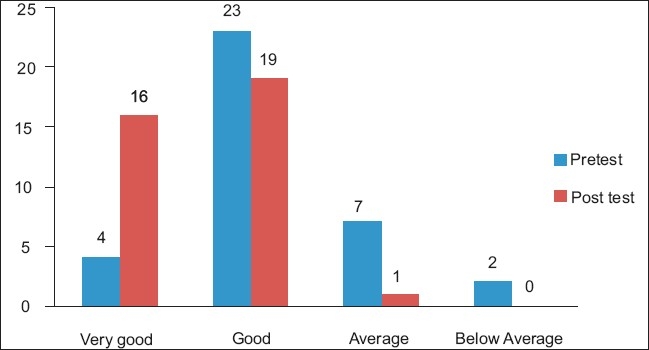
Frequency of the assessment score of pre and post test assessment scores

The IYCF program was conducted by the Gondia branch of BPNI at Gondia. A total of 36 female Anganawadi supervisor participants were registered and included in the study group. Before the IYCF training, 4(11.11%) the scores of the participants were as follows: very good, 23 (63.88%) good, 7 (19.44%) average, and 2 (5.55%) below average scores. The lowest pretest score was 11 (55%) and the highest was 18 (90%), which was out of a maximum score of 20. The lowest post-test score was 14 (70%) while the highest was 20 (100.0%), which was out of a maximum score of 20. Immediately after the IYCF training, 16 (44.44%) of the participants got very good scores, 19 (52.77%) got good, and 1 (2.77%) got an average score [Figure 1]. There were no below average scores in the post-test. Almost all (100%) of the supervisors, had the correct knowledge that breastfeeding should be started immediately, as early as possible after birth, breastfeeding should be continued as long as possible, baby should be kept rooming-in, complementary feeding should be started at six months of age, and bottle feeding should be totally avoided. The mean pretest score was 15.36 (SD 1.79), which had risen post-test to a mean of 17.16 (SD 1.59), and the calculated two-tailed *P* value suggested improvement in the post-workshop score, which was highly significant (*P* value < 0.000; 95% CI -2.46 to -1.14). The result also suggested that IYCF training had significantly improved the theoretical as well as practical knowledge on breastfeeding in the trainees. Figure 1 shows the distribution of the pre- and post-test scores of the supervisors.

## Discussion

AWWs are the key functionaries for effective implementation of Integrated Child Development Service Schemes (ICDS) in India. They are formally trained for non-formal, pre-school education of children between three and six years of age, primary health care and first-aid to children under six years and pregnant and nursing mothers, supplementary feeding of children of ages 0 - 6 years, referral services for severely malnourished children, and assisting health staff in immunization. A supervisor will provide continuous on-the-job guidance to AWWs, to bridge the gap between training and job requirement. Nutritional counseling of mothers of children aged 0 - 2 years is effective in positive behavioral modification and should be actively incorporated and emphasized. The risk of neonatal death is increased approximately four fold if milk-based fluids or solids are provided to breastfed neonates. The children need to be fed more and this can be achieved by counseling of mothers by AWWs, Auxiliary Nurse-Midwives, or supervisors. Social mobilization and community participation are critical for the success of any public health program.(4–6) In the present study, all the supervisors had an accurate knowledge that breastfeeding should be started as early as possible, immediately after birth. Similar findings have been reported among other health care personnel.(7–9) 94.44% had proper knowledge that prelacteal feed should not be given. Only 55.55% AWWs had knowledge that gripe water, ghutti, and honey were harmful to the baby. 66.11% of the supervisors knew that breastfeeding should be given on-demand, whereas, similar findings have been reported by other workers.(9–10) Satpathy *et al*.(11) conducted a study of AWWs on their knowledge, attitude, and practice surveys on breastfeeding, and reported that an average knowledge regarding breastfeeding was adequate. Twenty percent of those surveyed scored higher than 15; 74% scored between 10 and 15; and 6% scored less than 10 out of 20. However, in our study, a pretest revealed that 11.11% of the participants got very good scores, 63.88% got good, 19.44% got average, and 5.55% got below average score.

The simplest method of testing the effectiveness of training at a workshop is to administer the same set of objectively structured questions before and after the session and to analyze the difference. Halder *et al*.(12) reported that their training in infant feeding practices improved the knowledge of the participants and that repeat sessions were very useful. However, in our study, knowledge was assessed after IYCF event training in all health care workers and a significant difference was found in the post-test scores. The use of audiovisual aid increased the transfer of knowledge. Microteaching was the technique that could be exploited in peer groups or small batches to develop teaching and learning skills under the guidance of a supervisor. It was also a very good technique used in our training program to educate on breastfeeding. In our study, multimedia presentations were used for the lecture sessions and probably helped in a better transfer of knowledge.

## Conclusion

The Anganawadi supervisor gained the knowledge and skills regarding breastfeeding and complementary feeding after the training and there is need for in-service training of supervisor and other health workers/personnel, for updating their knowledge.
